# Higher preoperative Advanced Activities Score of the 2011 Knee Society Score is associated with greater patient satisfaction after ground kinematically aligned total knee arthroplasty

**DOI:** 10.1002/jeo2.70974

**Published:** 2026-10-29

**Authors:** Takuma Hayashi, Tomoyuki Matsumoto, Kemmei Ikuta, Masanori Tsubosaka, Tomoyuki Kamenaga, Yuichi Kuroda, Naoki Nakano, Kazunari Ishida, Shinya Hayashi, Ryosuke Kuroda

**Affiliations:** ^1^ Department of Orthopaedic Surgery Kobe University Graduate School of Medicine Kobe Japan; ^2^ Department of Orthopaedic Surgery Kobe Kaisei Hospital Kobe Japan

**Keywords:** ground mechanical axis, kinematic alignment, patient satisfaction, preoperative factors, total knee arthroplasty

## Abstract

**Purpose:**

Ground kinematically aligned total knee arthroplasty, which uses the ground mechanical axis as a reference, has shown favourable clinical outcomes. Nevertheless, preoperative factors associated with post‐operative satisfaction remain unclear. This study aimed to identify preoperative predictors of patient satisfaction after ground kinematically aligned total knee arthroplasty using patient‐reported outcome measures.

**Methods:**

A total of 117 consecutive patients with varus knee osteoarthritis who underwent primary ground kinematically aligned total knee arthroplasty were retrospectively analyzed. Patients were classified into excellent satisfaction (≥32 points) and suboptimal satisfaction (<32 points) groups based on the satisfaction subscale of the 2011 Knee Society Score. Stepwise multiple regression analysis was performed to identify preoperative factors associated with post‐operative satisfaction. Receiver operating characteristic curve analysis was used to determine the cut‐off value of the preoperative Advanced Activities Score for excellent satisfaction. Multivariable logistic regression analysis was performed to identify factors independently associated with excellent post‐operative satisfaction.

**Results:**

Preoperative Advanced Activities Score was significantly associated with post‐operative satisfaction (*B* = 0.057). Receiver operating characteristic analysis identified an optimal cut‐off value of 12 (sensitivity = 0.744; specificity = 0.677). Multivariable logistic regression confirmed that higher preoperative Advanced Activities Score was independently associated with excellent post‐operative satisfaction (odds ratio = 1.320; *p* < 0.001).

**Conclusions:**

Higher preoperative activity level was associated with greater post‐operative satisfaction after ground kinematically aligned total knee arthroplasty.

**Level of Evidence:**

Level III.

**Abbreviations**
AAS: Advanced Activities Score
AUC: area under the curve
BMI: body mass index
CI: confidence interval
FA: femoral angle
FKP: functional knee phenotype
FMA: femoral mechanical angle
HKA: hip–knee–ankle
ICC: intraclass correlation coefficient
KAM: knee adduction moment
KA‐TKA: kinematically aligned total knee arthroplasty
KSS: Knee Society Score
LDFA: lateral distal femoral angle
MA: mechanical axis
MA‐TKA: mechanically aligned TKA
MPTA: medial proximal tibial angle
nTA: navigation tibial angle
OA: osteoarthritis
PCL: posterior cruciate ligament
ROC: receiver operating characteristic
TA: tibial angle
TKA: total knee arthroplasty
TMA: tibial mechanical angle
VIF: variance inflation factor

## INTRODUCTION

Recent meta‐analyses have demonstrated that kinematically aligned total knee arthroplasty (KA‐TKA) provides superior functional outcomes compared with mechanically aligned TKA (MA‐TKA), although patient satisfaction appears comparable between alignment strategies [2, 10, 16, 25]. A previous study utilizing the 2011 Knee Society Score (KSS) suggested that patients with higher preoperative activity levels might be particularly suitable candidates for KA‐TKA because of greater post‐operative satisfaction [8, 17].

To further optimize kinematic alignment, ground KA‐TKA was developed, in which bone resection angles are determined according to the ground mechanical axis (MA). Ground MA is defined as the line connecting the centre of the hip joint and the plantar aspect of the calcaneus, passing through the centre of the knee joint in the native limb [15, 22]. As the ground MA may reflect a more balanced load distribution across the knee joint, full‐length lower‐limb assessment including the calcaneus has gained attention as an alternative method for evaluating lower‐extremity alignment [9, 23, 24]. Targeting a neutral ground MA has been demonstrated to provide more neutral weight‐bearing and a more equal coronal hindfoot plantar pressure distribution during walking [19]. Previous studies have demonstrated its feasibility and greater post‐operative range of motion than KA‐TKA [11, 12].

However, the preoperative factors associated with satisfaction after ground KA‐TKA remain unclear. The purpose of this study was to identify preoperative factors, including subscales of the 2011 KSS, that are associated with post‐operative patient satisfaction following ground KA‐TKA. It was hypothesized that a higher preoperative Advanced Activities Score (AAS), a subscale of the 2011 KSS, would be associated with greater post‐operative patient satisfaction. Identifying such predictive factors may facilitate preoperative patient counselling.

## MATERIALS AND METHODS

### Radiographic simulation for the ground KA‐TKA technique

To simulate ground KA‐TKA, distal femoral and proximal tibial resection lines were determined using standing full‐length coronal radiographs that included the calcaneus.

On the femoral side, distal femoral resection was simulated according to the calipered technique, with resection thicknesses of 9 mm laterally and 7 mm medially. Because the lateral cartilage was assumed to be preserved with a thickness of approximately 2 mm, a 9 mm lateral resection corresponded to a resection line 7 mm proximal to the radiographic lateral joint line. On the medial side, where the cartilage was assumed to be completely worn, a 7 mm resection was similarly represented by a resection line 7 mm proximal to the radiographic medial joint line. The femoral angle (FA) was defined as the angle between the femoral MA and a line perpendicular to the distal femoral resection line. In varus osteoarthritic knees, the FA generally represents a valgus angle (Figure 1a).

**Figure 1:**
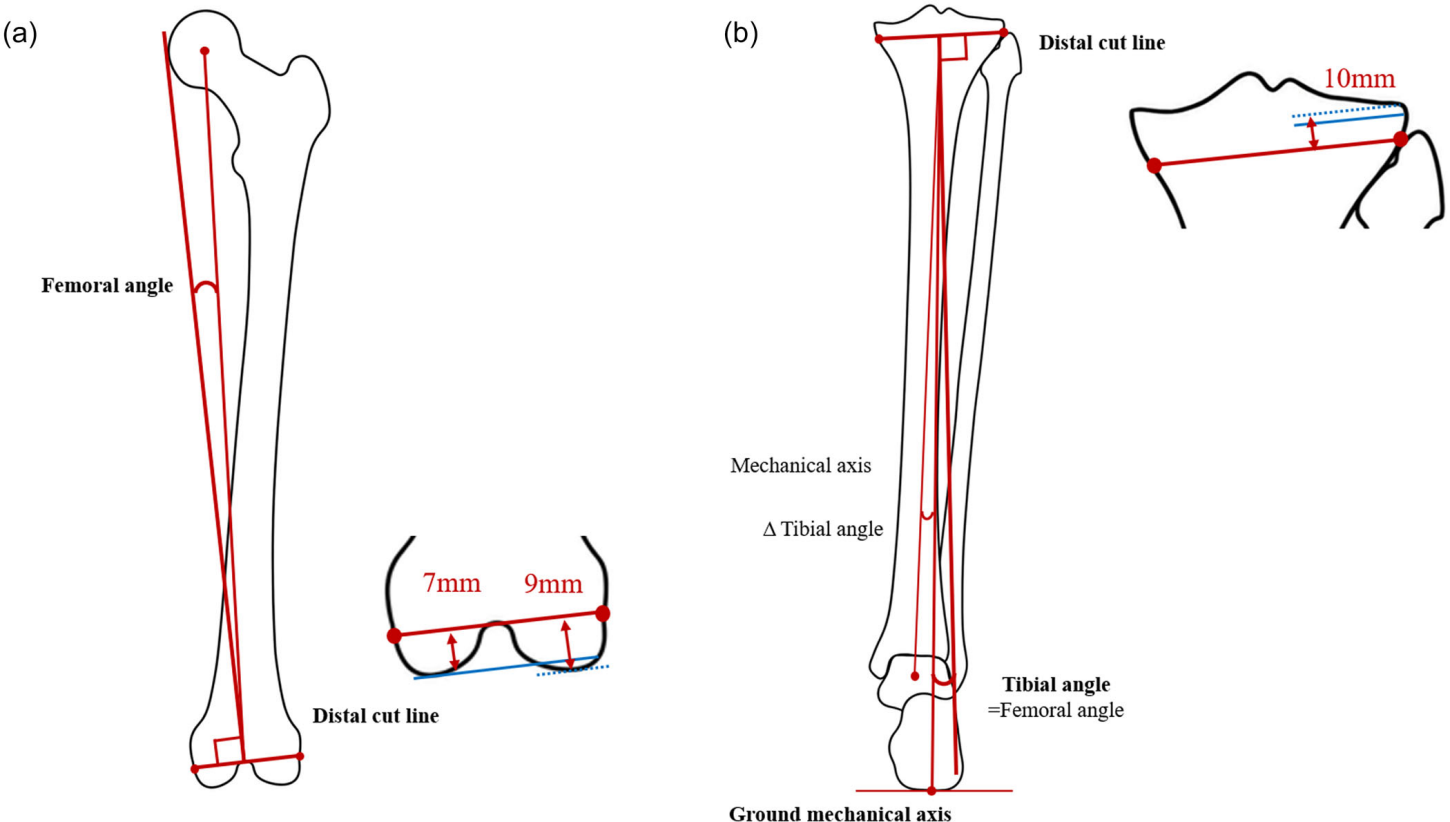
Radiographic simulation for the ground KA‐TKA technique. (a) Distal femoral bone cut simulation. The distal femoral resection was simulated according to the calipered technique, with resection thicknesses of 7 mm medially and 9 mm laterally. The femoral angle (FA) was defined relative to the femoral mechanical axis (MA). (b) Proximal tibial bone cut simulation. The proximal tibial resection line was adjusted to neutralize the FA relative to the ground MA, defined as the line connecting the centre of the proximal tibial resection line to the plantar aspect of the calcaneus. The difference between the conventional tibial MA and the ground MA was defined as the Δ tibial angle (ΔTA). KA‐TKA, kinematically aligned total knee arthroplasty.

On the tibial side, assuming a cartilage thickness of 2 mm, the proximal tibial resection line was positioned 8 mm distal to the lateral tibial joint line. The tibial resection line was then adjusted to neutralize the FA relative to the ground MA of the tibia, which was defined as the line connecting the centre of the proximal tibial resection line to the plantar aspect of the calcaneus (Figure 1b). Thus, when the FA was 3° of valgus, a tibial resection of 3° varus relative to the ground MA of the tibia was required. Because the navigation system references the ankle centre rather than the plantar aspect of the calcaneus, the angle between the conventional tibial MA and the ground MA was measured preoperatively and defined as ΔTA. Since the calcaneus is typically located lateral to the ankle centre, ΔTA generally represents a varus angle. The navigation tibial angle (nTA) was therefore calculated using the following equation: nTA (varus) = TA (varus) (= FA (valgus)) + ΔTA (varus) [11, 12].

### Radiographic evaluation

Preoperative and post‐operative radiographic parameters included the lateral distal femoral angle (LDFA), medial proximal tibial angle (MPTA) and hip–knee–ankle (HKA) angle. Functional knee phenotypes (FKPs) were classified preoperatively and post‐operatively based on the HKA, femoral mechanical angle (FMA) and tibial mechanical angle (TMA) [5, 6]. In addition, the ground MA ratio was evaluated post‐operatively. The ground MA ratio was defined as the point at which the line connecting the hip centre and the plantar aspect of the calcaneus intersected the tibial plateau, expressed as a percentage of the tibial plateau width.

Radiographic measurements were performed independently by two orthopaedic surgeons. Interobserver reliability for the LDFA, MPTA, HKA, FMA, TMA and ground MA ratio measurements was assessed using the intraclass correlation coefficient (ICC).

### Study design

The inclusion criteria were substantial pain and loss of function due to severe osteoarthritis (OA) of the knee (Kellgren–Lawrence Grade 3–4), with intercondylar osteophytes assessed using preoperative epicondylar‐view radiographs and Computed Tomography. Functional integrity of the PCL was confirmed intraoperatively before performing cruciate‐retaining TKA.

To make a fair assessment and minimize the influences of clinical variables, the exclusion criteria were knees with valgus deformity, severe varus deformity > 20°, flexion contracture > 20°, revision TKAs, active knee joint infections or the need for bilateral TKAs. To avoid compensatory hindfoot alignment change post‐operatively, patients with prior ankle or foot surgery, foot or ankle deformity (such as flat foot, hallux valgus and ankle OA), history of ankle fracture, and those unable to stand stably on one leg for >10 s without support were also excluded (Figure 2).

**Figure 2:**
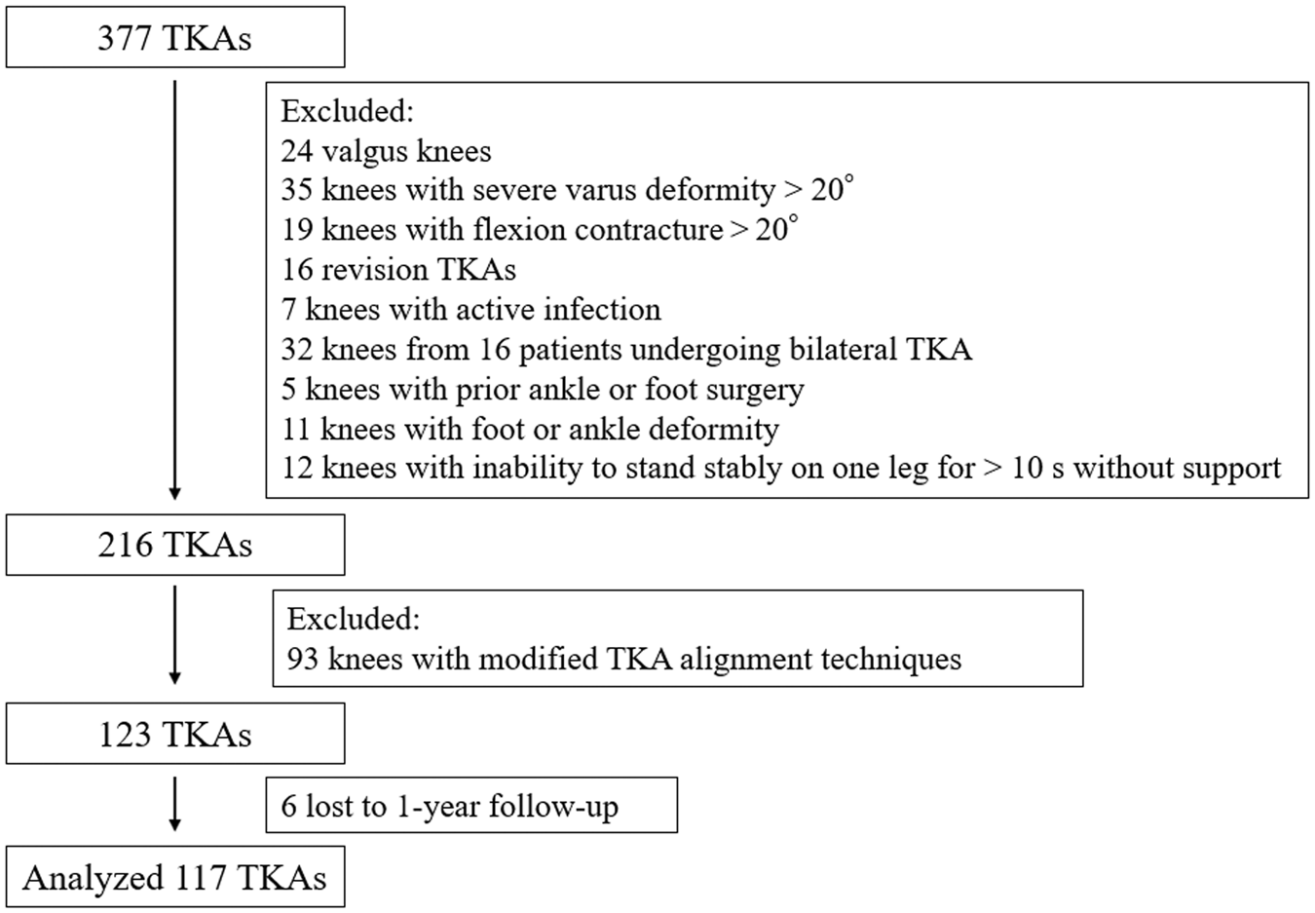
Patient flowchart. Flowchart showing the patient selection process and exclusion criteria. Of 377 TKAs initially assessed, 117 TKAs were included in the final analysis. TKA, total knee arthroplasty.

A total of 117 consecutive patients (98 women and 19 men) who underwent primary ground KA‐TKA for varus knee OA between May 2020 and January 2025 were included. The mean age was 75.5 ± 8.1 years, and the mean body mass index (BMI) was 26.8 ± 6.6 kg/m^2^. Of the 117 patients, 68 (57 women and 11 men) were implanted with the ATTUNE Knee System (DePuy Synthes, Johnson & Johnson), and 49 patients (41 women and 8 men) were implanted with the Persona Knee System (Zimmer Biomet). Navigation was performed using the iASSIST Knee System (Zimmer Biomet) in 49 cases and the KneeAlign 2 System (OrthAlign Inc.) in 68 cases. Implant type and navigation system corresponded completely: all 68 ATTUNE cases were performed using KneeAlign 2, whereas all 49 Persona cases were performed using iASSIST. All operative procedures were performed by a senior surgeon (T.M.) with >15 years of experience in performing TKAs.

### Operative procedures

All procedures were performed under air tourniquet control with the cuff inflated to 250 mmHg through a medial parapatellar approach. All surgeries were performed using the extension‐gap‐first technique. Following the confirmation of functional PCL based on intraoperative findings, the PCL insertion was preserved by creating a bony island.

For the ground KA‐TKAs, the distal femoral cut was performed according to the calipered method to reproduce the native distal femoral joint line while compensating for cartilage wear. The portable navigation system was subsequently used for the tibial cut to achieve the planned tibial resection angle based on the ground MA [7]. Before femoral osteotomies, minimum medial release, including osteophyte removal and release of the deep layer of the medial collateral ligament, was performed to maintain medial stability [14]. Femoral osteotomies were performed after correcting for cartilage wear from the distal and posterior femoral condyle, equal in thickness (9 mm) to the femoral component [13]. Femoral rotational angle was set at 0° relative to the posterior condylar axis [13]. Based on the FA value, which was confirmed by preoperative planning and the navigation system, the nTA was determined to target a neutral ground MA while preserving the original posterior tibial slope (up to 10°), as planned preoperatively. Thus, both distal femoral and proximal tibial resections were performed with reference to preoperative simulation and intraoperative measurements.

### Clinical evaluations

The 2011 KSS was assessed preoperatively and at 1 year post‐operatively. Patient demographics, radiographic findings, and clinical outcomes were retrospectively collected from electronic medical records and are summarized in Table 1.

**Table 1:** Comparison of preoperative and post‐operative clinical and patient‐reported outcome measures.

Variable	Preoperative	Post‐operative	Significance
Range of motion (°)			
Extension	−11.5 ± 6.0	−1.5 ± 2.9	<0.001
Flexion	122.1 ± 11.3	127.1 ± 8.4	<0.001
Radiographic parameters			
LDFA (°)	88.3 ± 1.4	88.5 ± 1.7	0.310
MPTA (°)	83.7 ± 2.3	87.8 ± 1.6	<0.001
HKA angle (°)	10.4 ± 3.8	0.7 ± 1.6	<0.001
Ground MA ratio (%)		50.2 ± 3.6	
2011 KSS			
Objective knee indicators	67.1 ± 8.0	95.2 ± 4.2	<0.001
Patient satisfaction	14.4 ± 6.0	28.5 ± 6.5	<0.001
Patient expectations	12.3 ± 2.4	11.5 ± 2.6	0.016
Functional activities	47.7 ± 16.5	77.5 ± 13.5	<0.001
Walking and standing	15.5 ± 6.6	23.9 ± 5.6	<0.001
Standard activities	15.8 ± 5.6	25.2 ± 4.1	<0.001
Advanced activities	9.8 ± 4.5	16.4 ± 5.2	<0.001
Discretionary knee activities	6.4 ± 3.0	11.9 ± 2.2	<0.001

*Note*: Data are presented as mean ± standard deviation.

Abbreviations: HKA, hip–knee–ankle; KSS, Knee Society Score; LDFA, lateral distal femoral angle; MA, mechanical axis; MPTA, medial proximal tibial angle.

The 2011 KSS comprises both patient‐reported and surgeon‐assessed components. The patient‐reported components comprise symptoms, patient satisfaction, patient expectations, and functional activities (walking and standing, standard activities, advanced activities and discretionary activities) [21]. The surgeon‐assessed components include alignment, stability and range of motion. The AAS, a subscale of the 2011 KSS functional activity score, consists of five items assessing higher‐level physical activities: climbing a ladder or step stool, carrying a shopping bag for approximately 200 m, squatting, kneeling and running. Each item is scored from 0 to 5, yielding a total score ranging from 0 to 25, with higher scores indicating a greater ability to perform advanced physical activities.

### Ethical approval

The hospital's ethics committee approved the study protocol (Kobe University, No. 290038). Written informed consent was obtained from all patients prior to participation.

### Statistical analysis

Normality of continuous variables was assessed using the Shapiro–Wilk test. Preoperative and post‐operative parameters were compared using paired Student's *t* tests for normally distributed variables and Wilcoxon signed‐rank tests for non‐normally distributed variables. For between‐group comparisons, Student's *t* test was used for normally distributed continuous variables and the Mann–Whitney *U* test for non‐normally distributed continuous variables. To identify factors associated with post‐operative patient satisfaction, stepwise multiple regression analysis was performed. The dependent variable was the post‐operative 2011 KSS satisfaction score. Independent preoperative variables included age, sex, BMI, knee extension and flexion angles, LDFA, MPTA, HKA angle, implant type and the preoperative 2011 KSS scores for objective knee indicators, patient satisfaction, expectations, walking and standing, standard activities, AAS and discretionary knee activities. To avoid perfect multicollinearity, implant type and navigation system were not simultaneously included in the regression model, and implant type was used as the representative variable. Multicollinearity among the candidate independent variables was assessed using the variance inflation factor (VIF), with a VIF < 5 considered to indicate the absence of problematic multicollinearity. A priori statistical power analysis was conducted using G*Power version 3.1.9.6 (Franz Faul, Universität Kiel) [4]. Assuming a significance level of *α* = 0.05, an effect size of 0.15, and a power of 0.95, the required sample size was calculated to be 83 patients.

In the Receiver operating characteristic (ROC) analysis, optimal outcomes were defined as ‘excellent patient satisfaction’ (post‐operative 2011 KSS satisfaction score ≥ 32), while scores < 32 were classified as ‘suboptimal’. This cut‐off has also been used in a previous study evaluating patient satisfaction after modified kinematically aligned TKA [8]. ROC curve analysis was performed to determine the optimal cut‐off value for AAS in predicting excellent patient satisfaction. An area under the curve (AUC) > 0.70 was considered acceptable. The optimal cut‐off value was determined using the Youden index [20]. Because the cut‐off value was derived from the same cohort, it was considered exploratory. Based on the ROC‐derived cut‐off, patients were divided into a high‐activity group (AAS ≥ 12) and a low‐activity group (AAS < 12). Post‐operative outcomes were compared between the two groups using unpaired Student's *t* tests for normally distributed variables and Mann–Whitney *U* tests for non‐normally distributed variables. Multivariable logistic regression analysis was performed using age, sex, BMI, implant type, knee extension and flexion angles, HKA angle, and the preoperative 2011 KSS objective knee indicators, patient satisfaction, and AAS as independent variables to identify factors associated with excellent patient satisfaction. All statistical analyses were performed using EZR software (version 1.65; Saitama Medical Centre, Jichi Medical University). A *p* value < 0.05 was considered statistically significant.

### Results

All values are presented as mean ± standard deviation. Preoperative and post‐operative clinical and radiographic outcomes are summarized in Table 1. The mean HKA angle improved significantly from 10.4 ± 3.8° preoperatively to 0.7 ± 1.6° post‐operatively (*p* < 0.001). The mean MPTA also changed significantly following surgery (*p *< 0.001). Functional knee phenotype analysis identified 32 distinct FKPs preoperatively and 13 post‐operatively. The neutral HKA–neutral FMA–neutral TMA phenotype was not observed preoperatively but was the most common phenotype post‐operatively, accounting for 56 of 117 knees (47.9%). The detailed distributions of preoperative and post‐operative FKPs are presented in Table S1. The post‐operative ground MA ratio was 50.2 ± 3.6%. Both knee extension and flexion angles improved post‐operatively. Furthermore, all subscales of the 2011 KSS except patient expectations demonstrated significant post‐operative improvement; patient expectations significantly decreased post‐operatively (*p* < 0.05). The ICCs for all radiographic measurements were ≥0.90, indicating excellent interobserver reliability.

Stepwise multiple regression analysis identified the preoperative AAS as the only significant predictor of post‐operative patient satisfaction (*B* = 0.057, *p* < 0.001; Table 2).

**Table 2:** Results of stepwise multiple regression analysis for predictors of post‐operative patient satisfaction.

Variable	Regression coefficient (*B*)	95% CI	SE	Significance
Advanced activities	0.057	0.037–0.078	0.010	<0.001

*Note*: Model *R* ^2^ = 0.208; adjusted *R* ^2^ = 0.201; *F* (1, 115) = 30.195; *p* < 0.001.

Abbreviations: B, unstandardized regression coefficient; CI, confidence interval; SE, standard error.

ROC curve analysis demonstrated that the preoperative AAS significantly predicted excellent post‐operative patient satisfaction, with an AUC of 0.713 (95% confidence interval [CI] = 0.599–0.826, *p* < 0.001), indicating acceptable discrimination ability (Figure 3). The optimal cut‐off value was 12 points, yielding a sensitivity of 74.4% and a specificity of 67.7%. This cut‐off was considered exploratory because it was derived from the same cohort. The continuous association between preoperative AAS and post‐operative 2011 KSS satisfaction score is shown in Figure 4.

**Figure 3:**
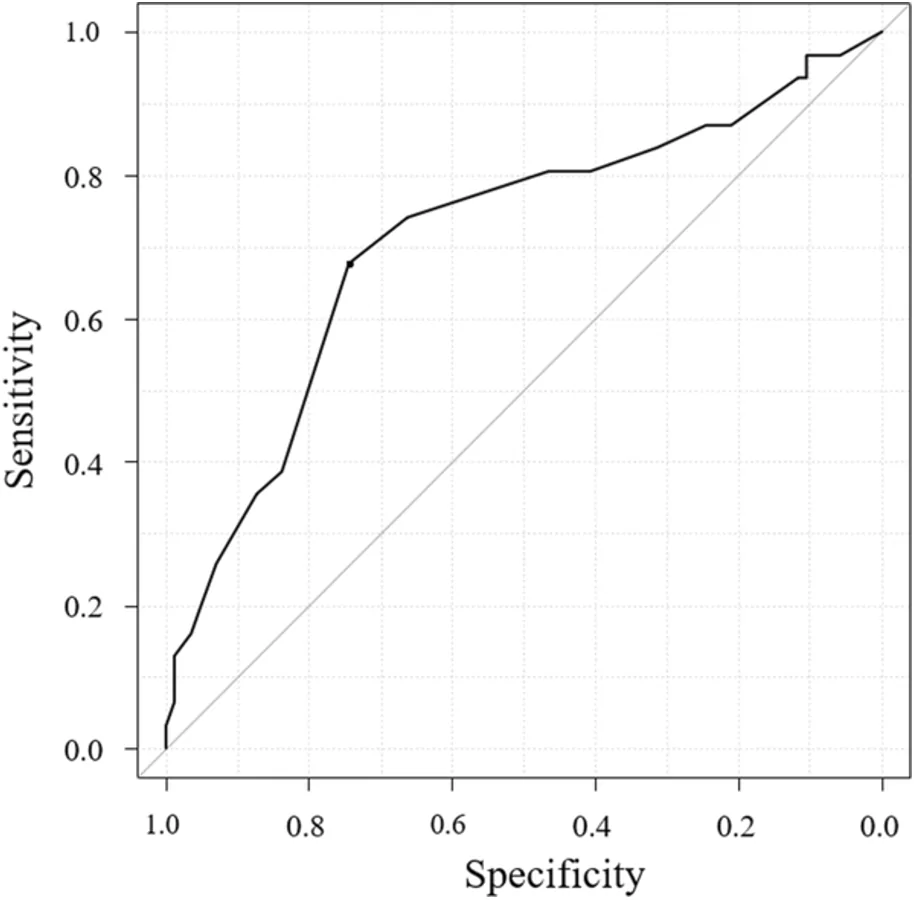
ROC curve of the preoperative Advanced Activities Score for predicting post‐operative patient satisfaction. AUC was 0.713 (95% CI = 0.599–0.826, *p* < 0.001), indicating acceptable discriminative ability. The optimal cut‐off value was 12, with a sensitivity of 0.744 and a specificity of 0.677. AUC, area under the curve; CI, confidence interval; ROC, receiver operating characteristic.

**Figure 4:**
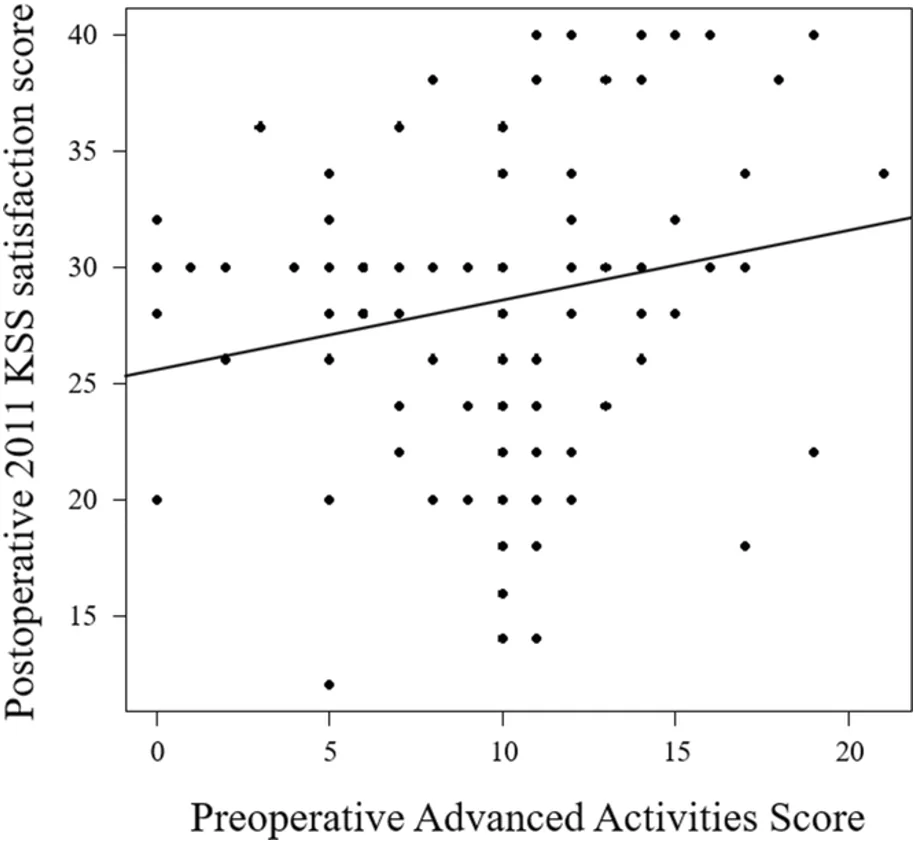
Association between preoperative Advanced Activities Score and post‐operative patient satisfaction. Scatter plot showing the continuous association between preoperative Advanced Activities Score and post‐operative 2011 Knee Society Score (KSS) satisfaction score. The solid line represents the fitted regression line.

Based on this threshold, patients were categorized into a high‐activity group (AAS ≥ 12; *n* = 31) and a low‐activity group (AAS < 12; *n* = 86). As shown in Figure 5, patients in the high‐activity group achieved significantly greater post‐operative satisfaction and better scores across all domains of the 2011 KSS than those in the low‐activity group. However, no significant differences were observed between the two groups in knee extension or flexion angles, LDFA, MPTA or HKA angle (Table 3).

**Figure 5:**
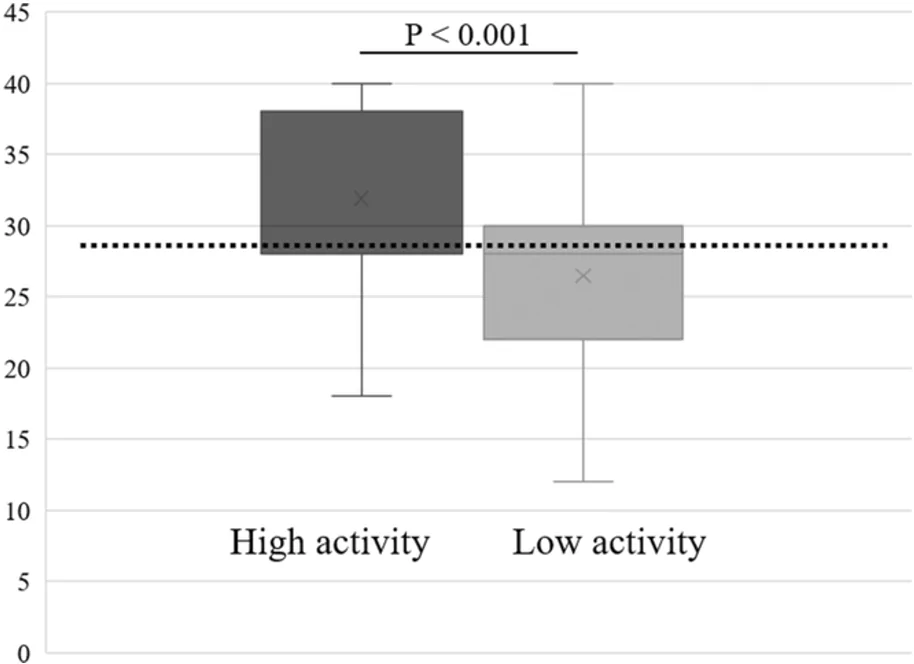
Post‐operative patient satisfaction stratified by preoperative activity level. Patients were divided into high‐activity (AAS ≥ 12, *n* = 31) and low‐activity (AAS < 12, *n* = 86) groups based on the receiver operating characteristic curve–derived cut‐off value. Box‐and‐whisker plots show significantly higher post‐operative patient satisfaction in the high‐activity group compared with the low‐activity group. The dashed line indicates the overall mean value. AAS, Advanced Activities Score.

**Table 3:** Comparison of post‐operative parameters between patients with high (*n* = 31) and low (*n* = 86) preoperative activity levels.

Variable	High activity group (*n* = 31)	Low activity group (*n* = 86)	Significance
Range of motion (°)			
Extension	−1.5 ± 2.3	−1.5 ± 3.2	0.961
Flexion	127.9 ± 7.9	126.7 ± 8.8	0.442
Radiographic parameters			
LDFA (°)	88.1 ± 1.6	88.7 ± 1.7	0.053
MPTA (°)	87.7 ± 1.4	87.9 ± 1.6	0.410
HKA angle (°)	0.5 ± 1.5	0.8 ± 1.7	0.384
Ground MA ratio (%)	50.0 ± 3.2	50.5 ± 4.2	0.496
2011 KSS			
Objective knee indicators	97.2 ± 2.7	94.0 ± 4.5	<0.001
Patient satisfaction	31.9 ± 6.0	26.5 ± 5.9	<0.001
Patient expectations	12.5 ± 2.3	10.9 ± 2.6	0.001
Functional activities	85.0 ± 9.6	73.2 ± 13.6	<0.001
Walking and standing	26.0 ± 4.4	22.6 ± 5.9	<0.001
Standard activities	27.0 ± 2.7	24.1 ± 4.4	<0.001
Advanced activities	19.3 ± 3.6	14.6 ± 5.2	<0.001
Discretionary knee activities	12.6 ± 1.8	11.4 ± 2.4	<0.001

*Note*: Data are presented as mean ± standard deviation.

Abbreviations: HKA, hip–knee–ankle; KSS, Knee Society Score; LDFA, lateral distal femoral angle; MA, mechanical axis; MPTA, medial proximal tibial angle.

Multivariable logistic regression analysis confirmed that the preoperative AAS was an independent predictor of excellent post‐operative patient satisfaction (odds ratio = 1.320, 95% CI = 1.130–1.530, *p* < 0.001; Table 4). Age, sex, BMI, implant type, knee extension angle, knee flexion angle, HKA angle, preoperative 2011 KSS objective knee indicators and preoperative patient satisfaction were not significantly associated with post‐operative patient satisfaction.

**Table 4:** Logistic regression analysis for predicting excellent patient satisfaction.

Variable	Odds ratio	95% CI	Significance
Age	1.010	0.945–1.070	0.836
Sex	0.401	0.099–1.620	0.199
BMI	1.030	0.965–1.100	0.358
Implant type	0.566	0.212–1.510	0.255
Range of motion (°)			
Extension	1.050	0.933–1.180	0.423
Flexion	0.955	0.909–1.000	0.073
Radiographic parameters			
HKA angle (°)	1.040	0.934–1.170	0.457
2011 KSS			
Objective knee indicators	0.952	0.893–1.010	0.124
Patient satisfaction	0.965	0.879–1.060	0.464
Advanced activities	1.320	1.130–1.530	<0.001

Abbreviations: BMI, body mass index; CI, confidence interval; HKA, hip–knee–ankle; KSS, Knee Society Score.

## DISCUSSION

The most important finding of the present study was that preoperative activity level, as assessed by the AAS of the 2011 KSS, was significantly associated with post‐operative patient satisfaction following ground KA‐TKA. In particular, patients with higher preoperative activity levels achieved greater post‐operative satisfaction following this technique.

Although several meta‐analyses and systematic reviews have compared KA‐TKA with MA‐TKA, their findings remain inconsistent. Some studies have demonstrated superior clinical and functional outcomes following KA‐TKA, whereas others have reported no significant differences between alignment strategies [1, 2, 3, 25]. Consequently, identifying patient characteristics associated with favourable outcomes after KA‐TKA has become increasingly important. Previous studies have suggested that KA‐TKA might be particularly beneficial for younger, highly active male patients [17]. In contrast, in the present study, neither age nor sex was significantly associated with post‐operative patient satisfaction. This discrepancy may be related to differences in patient demographics. Compared with the previous report, the present cohort was older (mean age: 75.5 ± 8.1 years vs. 73.4 ± 8.5 years) and included a lower proportion of male participants (16% vs. 25%), which may have attenuated the influence of age and sex on post‐operative outcomes.

Among the various preoperative factors evaluated, activity level was the only factor significantly associated with post‐operative satisfaction. This finding is consistent with previous reports highlighting the importance of preoperative activity level in patients undergoing KA‐TKA. Ikuta et al. reported that the preoperative AAS was associated with post‐operative satisfaction after modified KA‐TKA and identified a cut‐off value of 8 points [8]. In contrast, the present study identified a higher threshold of 12 points for ground KA‐TKA. The difference in cut‐off values may partly reflect differences in baseline activity levels between the study populations (mean preoperative AAS: 9.8 ± 4.5 vs. 8.0 ± 6.0). Therefore, the cut‐off value of 12 points in the present cohort should be interpreted as exploratory.

Niki et al. demonstrated that the joint line obliquity achieved by KA‐TKA reduced the peak knee adduction moment (KAM) during gait despite slight residual varus limb alignment, suggesting that the reduction in KAM allows constitutional varus alignment to be better tolerated [18]. Furthermore, they proposed that patients with high preoperative activity levels and a narrow step width may be particularly suitable candidates for KA‐TKA [17]. The ground MA has been proposed to improve the parallelism between the joint line and the ground, thereby promoting a more neutral weight‐bearing alignment [17, 23].

Ground KA‐TKA differs from the other KA‐TKA techniques in that alignment is determined according to the ground MA, defined by the hip centre and the bottom of the calcaneus. This approach aims to restore a more physiological lower‐limb alignment and knee kinematics. A previous investigation has demonstrated that ground KA‐TKA achieves greater post‐operative range of motion than modified KA‐TKA while maintaining comparable overall clinical outcomes [11]. However, whether these characteristics influence the association between preoperative activity level and post‐operative satisfaction remains unclear.

In the present study, preoperative AAS ≥ 12 was identified as an exploratory threshold associated with excellent post‐operative patient satisfaction. An AAS of 12 (maximum score, 25) reflects the combined performance of five different activities and does not represent the ability to perform any single specific task. From a clinical perspective, this finding may provide useful information for preoperative counselling. Given that this cut‐off was derived and evaluated within the same cohort, it should not be considered a clinical threshold for patient selection without further validation.

Several limitations should be acknowledged. First, the sample size was relatively small, and patients with valgus or severe varus deformities were excluded, potentially limiting the applicability of the findings to such deformities. Second, the follow‐up period was limited to 1 year, and long‐term outcomes, including implant survivorship, were not evaluated. Third, all procedures were performed by a single experienced surgeon, which may limit generalizability of the results. Fourth, two different implant systems and navigation systems were used. Implant type and navigation system were completely matched. Therefore, their individual effects on post‐operative outcomes could not be distinguished. Fifth, the sample size was relatively limited for the multivariable analysis, and the possibility of model overfitting cannot be excluded. Finally, the AAS cut‐off of 12 was derived and evaluated within the same cohort and should therefore be considered exploratory. External validation in an independent cohort is required before this cut‐off can be applied clinically. Future multicenter studies involving larger patient populations, multiple implant designs and navigation systems, and longer follow‐up periods are warranted to validate these findings and to determine whether the observed association between preoperative activity level and post‐operative satisfaction is specific to ground KA‐TKA.

## CONCLUSIONS

Higher preoperative activity level was associated with greater post‐operative satisfaction after ground KA‐TKA.

## AUTHOR CONTRIBUTIONS

Takuma Hayashi contributed to the study conception and design, data collection, statistical analysis, interpretation of the data and manuscript drafting. Tomoyuki Matsumoto supervised the study and critically revised the manuscript. All authors contributed to data interpretation, reviewed the manuscript and approved the final version.

## FUNDING INFORMATION

The authors have no funding to report.

## CONFLICT OF INTEREST STATEMENT

The authors declare no conflicts of interest.

## ETHICS STATEMENT

This study was approved by the Institutional Review Board of Kobe University Graduate School of Medicine (Approval No. 290038). Written informed consent was obtained from all participants prior to surgery.

## Supporting information

Supporting File 1.

## Data Availability

The data that support the findings of this study are available from the corresponding author upon reasonable request.
