# Supplementary material for: Higher preoperative Advanced Activities Score of the 2011 Knee Society Score is associated with greater patient satisfaction after ground kinematically aligned total knee arthroplasty
Source: J Exp Orthop. 2026 Oct 29;13(4):e70974. doi: 10.1002/jeo2.70974 (PMC13718614; doi:10.1002/jeo2.70974)
Supplement: Supplementary file 1 [file JEO2-13-e70974-s001.docx]

**Supplementary Table 1. Distribution of preoperative and postoperative functional knee phenotypes**

| Preoperative FKPs | |  |  |  |
| --- | --- | --- | --- | --- |
| **HKA** | **FMA** | **TMA** | **n** | **%** |
| VAR 3 | NEU | NEU | 2 | 1.7 |
| VAR 3 | NEU | VAR 3 | 5 | 4.3 |
| VAR 3 | VAR 3 | VAR 3 | 1 | 0.9 |
| VAR 6 | NEU | NEU | 4 | 3.4 |
| VAR 6 | NEU | VAR 3 | 5 | 4.3 |
| VAR 6 | NEU | VAR 6 | 3 | 2.6 |
| VAR 6 | VAR 3 | NEU | 1 | 0.9 |
| VAR 6 | VAR 3 | VAR 3 | 1 | 0.9 |
| VAR 6 | VAR 6 | NEU | 1 | 0.9 |
| VAR 9 | NEU | NEU | 7 | 6.0 |
| VAR 9 | NEU | VAR 3 | 14 | 12.0 |
| VAR 9 | NEU | VAR 6 | 6 | 5.1 |
| VAR 9 | VAR 3 | NEU | 6 | 5.1 |
| VAR 9 | VAR 3 | VAR 3 | 5 | 4.3 |
| VAR 9 | VAR 3 | VAR 9 | 1 | 0.9 |
| VAR 9 | VAR 6 | VAR 3 | 1 | 0.9 |
| VAR 12 | NEU | NEU | 3 | 2.6 |
| VAR 12 | NEU | VAR 3 | 9 | 7.7 |
| VAR 12 | NEU | VAR 6 | 6 | 5.1 |
| VAR 12 | VAR 3 | NEU | 3 | 2.6 |
| VAR 12 | VAR 3 | VAR 3 | 3 | 2.6 |
| VAR 12 | VAR 3 | VAR 6 | 2 | 1.7 |
| VAR 15 | NEU | VAR 3 | 4 | 3.4 |
| VAR 15 | NEU | VAR 6 | 6 | 5.1 |
| VAR 15 | VAR 3 | VAR 3 | 7 | 6.0 |
| VAR 15 | VAR 3 | VAR 6 | 2 | 1.7 |
| VAR 15 | VAR 3 | VAR 9 | 1 | 0.9 |
| VAR 18 | NEU | VAR 6 | 1 | 0.9 |
| VAR 18 | VAR 3 | VAR 3 | 3 | 2.6 |
| VAR 18 | VAR 3 | VAR 6 | 2 | 1.7 |
| VAR 18 | VAR 3 | VAR 9 | 1 | 0.9 |
| VAL 3 | NEU | NEU | 1 | 0.9 |
|  |  |  |  |  |
| Postoperative FKPs | |  |  |  |
| **HKA** | **FMA** | **TMA** | **n** | **%** |
| NEU | NEU | NEU | 56 | 47.9 |
| NEU | NEU | VAR 3 | 1 | 0.9 |
| NEU | NEU | VAL 3 | 3 | 2.6 |
| NEU | VAR 3 | NEU | 6 | 5.1 |
| NEU | VAR 3 | VAL 3 | 16 | 13.7 |
| VAR 3 | NEU | NEU | 1 | 0.9 |
| VAR 3 | NEU | VAR 3 | 1 | 0.9 |
| VAR 3 | VAR 3 | NEU | 17 | 14.5 |
| VAR 3 | VAR 3 | VAR 3 | 1 | 0.9 |
| VAR 3 | VAR 3 | VAL 3 | 6 | 5.1 |
| VAL 3 | NEU | VAL 3 | 6 | 5.1 |
| VAL 3 | VAR 3 | VAL 3 | 2 | 1.7 |
| VAL 3 | VAL 3 | NEU | 1 | 0.9 |

HKA: hip–knee–ankle; FMA: femoral mechanical angle; TMA: tibial mechanical angle; FKP: functional knee phenotype.

**Table legends**

**Supplementary Table 1. Distribution of preoperative and postoperative functional knee phenotypes**

HKA: hip–knee–ankle; FMA: femoral mechanical angle; TMA: tibial mechanical angle; FKP: functional knee phenotype.
